# Pathogenicity and Rapid Growth Kinetics of Feline Immunodeficiency Virus Are Linked to 3′ Elements

**DOI:** 10.1371/journal.pone.0024020

**Published:** 2011-08-26

**Authors:** Jesse Thompson, Martha MacMillan, Karen Boegler, Charles Wood, John H. Elder, Sue VandeWoude

**Affiliations:** 1 Department of Microbiology, Immunology, and Pathology, Colorado State University, Fort Collins, Colorado, United States of America; 2 Department of Immunology and Microbial Science, The Scripps Research Institute, La Jolla, California, United States of America; 3 School of Biological Sciences-Nebraska Center for Virology, University of Nebraska, Lincoln, Nebraska, United States of America; Food and Drug Administration, United States of America

## Abstract

Chimeric viruses constructed between a highly pathogenic Feline Immunodeficiency Virus isolate (FIV-C36) and a less pathogenic but neurotropic strain (FIV-PPR) have been used to map viral genetic determinants of *in vivo* pathogenicity. Chimeric virus FIV-PCenv, which contains FIV-C36 genome from the 3′ region of *pol* to upstream of the 3′LTR on an FIV-PPR backbone, was previously shown to be replication-competent *in vivo*, inducing altered CD4^+^ T-cell and neutrophil profiles intermediate between parental strains following a delay in viral replication during initial infection. Examination of FIV-PCenv proviral sequences recovered at week 11 post-infection revealed two changes compared to initial viral inoculum; the most significant being arginine to histidine in the integrase region of Pol at residue 813 (R813H). Pooled plasma from the initial *in vivo* study was used to inoculate a second cohort of cats to determine whether similar virulence and kinetics could be established following primary infection. Viral replication kinetics and immunocyte profiles were monitored in blood, bone marrow, and saliva over a one-year period. Passaged FIV-PCenv again displayed intermediate phenotype between parental strains, but unlike primary experiments, the onset of acute viremia was not delayed. CD4/8 alterations were noted in all groups of animals, though significant changes from controls were delayed in FIV-PPR infected animals compared to FIV-C36 and FIV-PCenv. *In vivo* passage of FIV-PCenv increased replication-competence relative to the initial molecularly-cloned chimera in association with one adaptive nucleotide change in the 5′ end of the genome relative to primary tissue culture inoculum, while mutations in the 3′ end of the genome were not detected. The results are consistent with the interpretation that 3′ elements contribute to heightened virulence of FIV-C36, and that integrase residue 813 plays an important role in facilitating successful *in vivo* replication.

## Introduction

Feline immunodeficiency virus (FIV) is a naturally-occurring lentivirus of domestic cats. Infection results in acquired immunodeficiency syndrome associated with progressive loss of CD4^+^ T-lymphocytes [1], [2], [3], [4], [5], [6]. FIV has a similar genome structure as human immunodeficiency virus (HIV) [7], containing several open-reading-frame (ORF) accessory genes [8], [9], and also uses a two-receptor mechanism, with cellular entry via CXCR4 chemokine receptor [10], [11], [12], [13]. Similarities between these complex lentiviruses make FIV infections a relevant animal model for studies of HIV-AIDS [14], [15].

**Figure 1:**
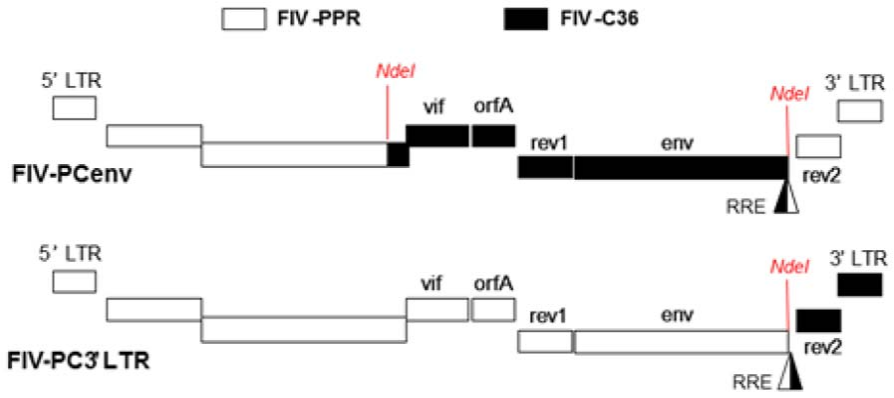
Clade A/C FIV chimeras. Clade C FIV-C36 sequences are depicted in black, and clade A FIV-PPR sequences in white. Recombinant clone FIV-PCenv was generated by exchanging ∼5 kb of FIV-C36 between the NdeI restriction endonuclease recognition sites. FIV-PC3′LTR contains FIV-C36 3′LTR including the second exon of *rev*.

Five FIV clades (A–E) have been identified and are distinguished by envelope sequence [16], [17], [18], [19], [20], [21]. Two isolates, FIV-PPR and FIV-CPG (molecular clone FIV-C36), belonging to clades A and C, respectively, are variable with regard to disease potential [4], [22], [23]. Pathology of lentivirus subtypes can be attributed to any number of properties, including replication rates or levels dictated by a combination of viral and host factors; these include viral genome secondary structure, efficacy of evasion of a host innate or adaptive immune response, binding affinity to cell surface receptors, and epigenetic factors. While it is probable that host innate and adaptive immune responses relating to host genotype contribute to these differences, pathology of FIV-C36 and molecularly-cloned FIV-PPR is predictable from study to study [6], [23], [24]. Chimeric viruses constructed between these two phenotypically distinct strains of FIV are potentially useful tools to identify viral molecular determinants of virulence and/or differences in viral tropism or kinetics. Several chimeric constructs were therefore developed by exchanging elements between FIV-C36 and FIV-PPR as previously reported and reviewed in Figure 1 [24].

Initial *in vivo* studies demonstrated that FIV-PC3′LTR (FIV-C36 *rev*2 and 3′ LTR on the FIV-PPR backbone) was infectious, although attenuated compared to parental constructs. In contrast, FIV-PCenv displayed a phenotype intermediate to parental viruses with regard to plasma viremia, proviral load, and CD4^+^ T-cell and neutrophil declines. This construct includes FIV-C36 *vif*, *orfA*, *env*, and the first exon of *rev* on the FIV-PPR backbone; approximately 250 nucleotides of 3′ FIV-C36 *pol* are also included, resulting in a chimeric integrase protein. Interestingly, FIV-PCenv peak viral load and development of clinical disease was delayed by three weeks compared to FIV-PPR, FIV-PC3′LTR, and FIV-C36, despite equivalent challenge doses [24]. These findings suggested that (1) FIV-C36 genomic elements represented in FIV-PCenv are related to pathogenicity, and (2) infection with primary FIV-PCenv may have resulted in accumulation of mutations leading to enhanced replication capacity following an initial delay. Increases in pathogenicity of HIV/SIV (SHIV) chimeras have been reported during *ex vivo* [25], [26] and *in vivo* passages in macaques [27]. Further, quasispecies diversity with respect to receptor usage and host-cell range are broadened upon viral passage [28], supporting the hypothesis that FIV-PCenv was similarly affected.

**Figure 2:**
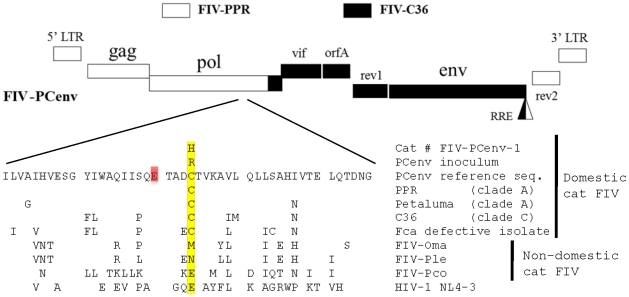
Amino acid comparison of sequences from cat FIV-Pcenv-1 and virus inoculum with parental clones (PPR and C36), another clade A domestic cat sequence (Petaluma), and nondomestic feline species (FIV-Oma = Pallas cat; FIV-Ple = lion; FIV-Pco-puma). FIV-Fca defective isolate indicates replication-deficient strain from a domestic cat. Highlighted cysteines are conserved among domestic cat, but variation is noted at this site between and among felid species, and in prototypic HIV-1 lab strain NL4-3. Initial viral inoculum had an arginine incorporated at this site, which was replaced by histidine following infection of cat # FIV-PCenv-1.

We investigated whether delayed pathogenicity of FIV-PCenv was an inherent phenotype of its manipulated genome, or if elements near the *env* region contribute to enhanced virulence after a period of adaptation. To test the hypothesis that FIV-PCenv acquired mutations during primary infections, we performed sequence analysis of proviral DNA from an FIV-PCenv infected cat during peak viremia. In addition, a second cohort of domestic cats was inoculated with pooled plasma from the primary FIV-PCenv and parental strain infections to evaluate the impact of serial passage on viral replication kinetics and pathogenicity. In this year-long *in vivo* analysis, peripheral and bone marrow viral kinetics, immunopathogenicity, and viral salivary excretion were evaluated. While no extensive nucleotide substitutions were detected in the 3′ end of the chimeric genome following *in vivo* passage, two nucleotide changes were detected during comparisons of *in vitro* inoculum and *in vivo* derived sequences in the 5′ half of FIV-PCenv. A silent T→C mutation was found at amino acid 64 of Gag, resulting in an ATT→ATC codon change in isoleucine. A more significant change was an arginine to histidine substitution at residue 813 (R813H) within the integrase region of *pol*. This residue has been reported as a cysteine in parental FIV-PPR (and was verified as such in this study) as well as all other domestic FIV sequences; however, variations occur amongst the non-domestic FIVs and the homologous site in HIV-1 integrase (Figure 2). Thus, a substitution of Cys>Arg apparently occurred during *in vitro* propagation. Secondary *in vivo* passage of chimeric virus using plasma from primary infections, during which a further mutation of Arg 813 to His occurred, resulted in more rapid viral kinetics compared to parental strains. FIV-PCenv viremia in bone marrow, plasma, and saliva ultimately plateaued in the same range observed in cats infected with FIV-C36, surpassing FIV-PPR levels. FIV-PCenv plasma RNA along with PBMC and bone marrow proviral levels were elevated during serial passage when compared to primary infection, an effect not observed in cats infected with parental viruses. Further analysis of site 813 in proviral and plasma isolates from all cats in the primary passage indicated nonsynonymous mutations at this site in 4/5 cats relative to viral inoculate (2 animals with R813H and 2 with R813C). All 5 cats in the secondary passage had a His residue at this site, consistent with the predominant plasma viral quasispecies in the 2^nd^ passage inoculum. These findings suggest that: (1) serial passage with a chimeric virus containing 3′ elements of virulent FIV-C36 confers enhanced replication capacity to FIV-PPR in both peripheral and parenteral compartments; and, (2) changes in replication rates *in vivo* between the primary and secondary passages were a consequence of a mutation at residue 813 of integrase. Since the chimeric virus included portions of 3′ *pol* from FIV-PPR and FIV-C36, this may represent a requirement for coordination of this residue with components of the C-terminus of the integrase protein.

## Results

### FIV-PCenv sequence analysis

We chose the time point at which group mean levels during primary infection were highest (day 77 PI) for amplification and analysis of provirus from an FIV-PCenv infected cat [24]. This sample was chosen because the likelihood of detecting nucleotide differences compared to inoculum would be greatest after the initial lag period displayed by the chimera. Complete proviral sequence was characterized from Cat# FIV-PCenv-1 at a timepoint when 1.68×10^4^ FIV proviral equivalents per million PBMC was detected. Consensus sequence of six clones per region amplified was aligned with that of virus stock sequenced using identical methods.

**Figure 3:**
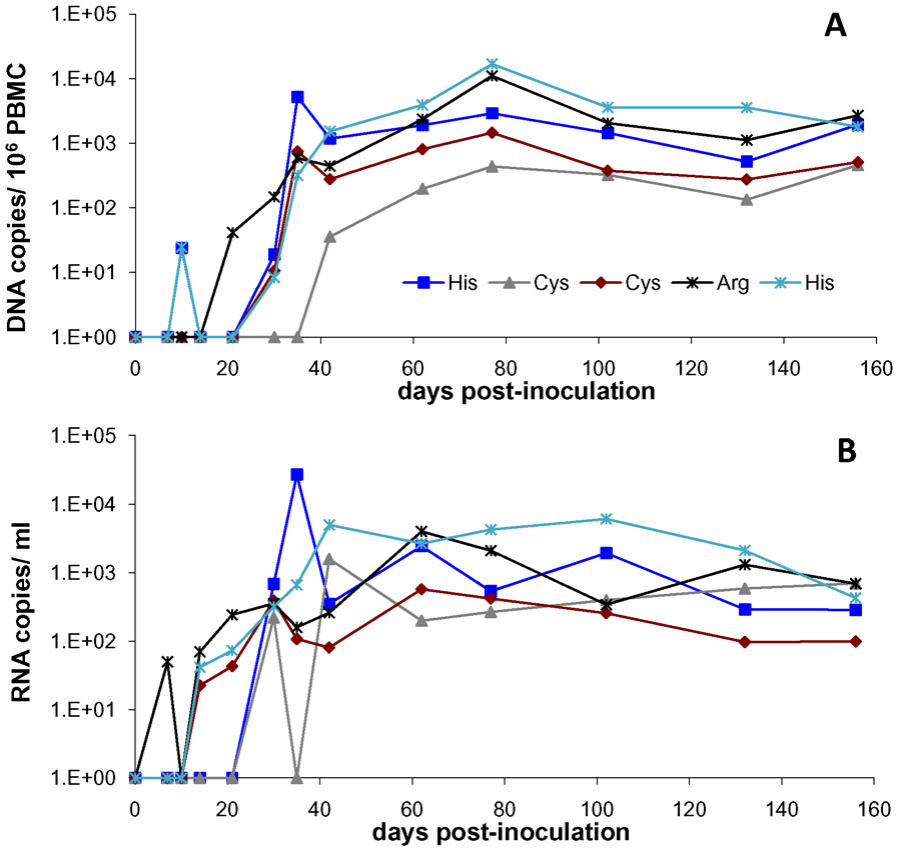
Individual cats' PBMC (A) and plasma (B) viral loads during infection with FIV-PCenv virus stock. Direct sequencing of integrated and circulating virus at days 102 and 156 post-inoculation detected polymorphism at Pol residue 813 (integrase).

Two changes were observed between provirus obtained from Cat # FIV-PCenv-1 and FIV-PCenv stock. First, a silent T→C mutation was found at amino acid 64 of Gag, resulting in an ATT→ATC codon change in isoleucine. Second, a mutation at amino acid 813 of Pol resulted in a codon change from CGT (arginine) in inoculum to CAT (histidine) in Cat # FIV-PCenv-1 (R813H; Figure 2). Original FIV isolates PPR and C36, as well as two other FIV isolates (clade A isolate Petaluma and defective *Felis catus* strain reported in GenBank), have cysteine at this position, which falls within the integrase region of Pol; sequence analysis verified that the molecular clone of FIV-PPR used to generate FIV-PCenv contained Cys at this site. Alignment with nondomestic FIV and HIV sequences demonstrates divergence at this residue among more distantly related FIVs (Figure 2). To determine whether virus from the remaining FIV-PCenv infected cats harbored this mutation in integrase, we directly sequenced both integrated (days 102 and 156 PI) and circulating virus (day 77 PI), and found the same nonsynonomous mutation to His in a second individual (FIV-PCenv-5), reversion to wild-type Cys in two cats (FIV-PCenv-3 and 4), and sequence consistent with the inoculum (Arg) in the remaining animal (FIV-PCenv-2; Table 1). Figure 3 shows PBMC (A) and plasma (B) viral loads of individual FIV-PCenv infected cats, in which a His mutation tended to display higher viremia compared to Cys or Arg. Additionally, all cats inoculated with pooled plasma from the first inoculation were infected with virus containing the CAT His codon at the first timepoint (day 21 PI) in which there was detectable viremia in all individuals (Table 1).

**Table 1:** Analysis of Phases I and II circulating and integrated FIV-PCenv integrase residue 813.

	codon/aa		
pFIV-PPR reference*	TGT/Cys		
pFIV-C36 reference*	TGT/Cys		
FIV-PCenv inoculum**	CGT/Arg		
FIV-PCenv-1 (d77)**	CAT/His		

Phase I	d77 plasma	d102 PBMC	d156 PBMC
FIV-PCenv -1	CAT/His	CAT/His	CAT/His
FIV-PCenv -2	CGT/Arg	CGT/Arg	CGT/Arg
FIV-PCenv -3	TGT/Cys	TGT/Cys	TGT/Cys
FIV-PCenv -4	NA	TGT/Cys	TGT/Cys
FIV-PCenv -5	CAT/His	CAT/His	CAT/His

Phase II	d21 plasma	d21 PBMC	
FIV-PCenv -6	CAT/His	CAT/His	
FIV-PCenv -7	CAT/His	CAT/His	
FIV-PCenv -8	CAT/His	CAT/His	
FIV-PCenv -9	CAT/His	CAT/His	
FIV-PCenv -10	CAT/His	CAT/His	

*sequence of parental plasmids used for cloning FIV-PCenv confirmed at TSRI.

**TOPO clones from full-genome sequencing of Phase I inoculum and one individual cat.

### *In vivo* passage of FIV-PCenv

**Figure 4:**
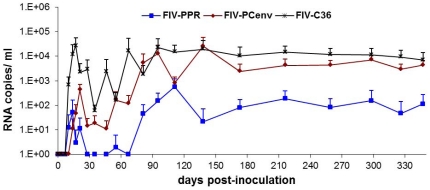
Circulating virus during *in vivo* passage of clade A FIV-PPR, clade C FIV-C36, and A/C chimeric FIV-PCenv. Group means with standard deviation for plasma viremia are shown as numbers of RNA copies/ml over the course of the study. PCR reactions were performed in triplicate. Mock-infected cats had undetectable viremia (data not shown).

To test if delayed viral loads noted during first round infections of domestic cats [24] were indicative of an initial period of virus adaptation that could be overcome with serial passage, pooled plasma from infected cats was used as inoculum for second round *in vivo* infections. Five cats per group were inoculated intravenously with plasma containing either FIV-PCenv, FIV-C36, or FIV-PPR, or naïve plasma at doses equalized as described in Methods. Relative levels of viremia were then assessed over time by measuring both the number of viral RNA copies per milliliter plasma and the number of viral DNA copies detected per 10^6^ PBMC DNA equivalents. Initial spikes of viral RNA copies/ml occurred 17–21 days PI for all infection groups (Figure 4) and were highest in FIV-C36, followed by FIV-PCenv, then FIV-PPR. Circulating virus levels fluctuated for a period after this initial spike for all groups, with most FIV-PPR values below the level of detection until day 81 PI. In fact, two of five FIV-PPR infected cats were aviremic for nearly six months, until day 173 PI. Maximum mean levels of plasma viremia were detected between three and five months PI for all groups, remaining relatively constant until day 347 PI and were as follows (RNA copies/ml plasma): FIV-PPR = 5.68×10^2^, FIV-PCenv = 2.58×10^4^, and FIV-C36 = 2.26×10^4^ (Figure 4).

**Figure 5:**
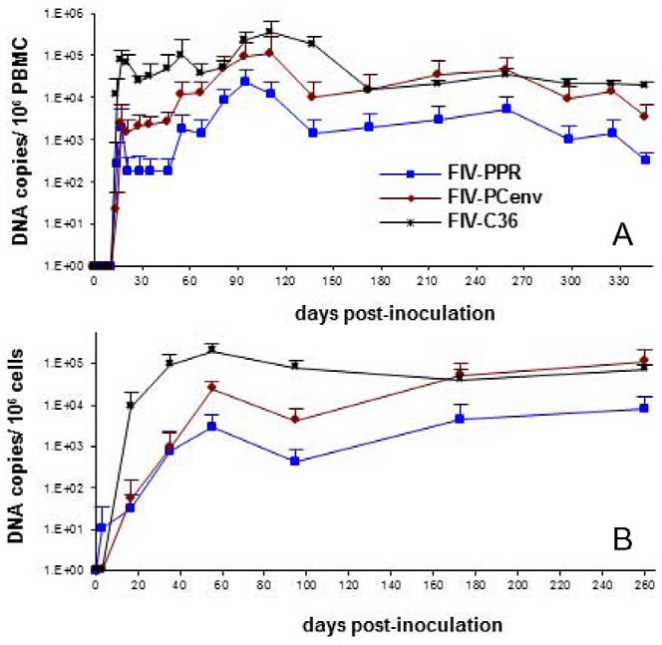
PBMC (A) and bone marrow (B) proviral loads during *in vivo* passage of FIV-PPR, FIV-C36, and FIV-PCenv. Group means with standard deviation are shown as numbers of FIV gag equivalents/10^6^ cells over the course of the study. PCR reactions were performed in triplicate. Mock-infected cats were provirus-negative (data not shown).

FIV-PCenv proviral kinetics in PBMC or bone marrow were similar to circulating virus following *in vivo* passage. All animals had detectable integrated proviral *gag* sequences in PBMCs on day 14 and values were approximately equivalent for all constructs on day 17 (Figure 5A). After day 17, FIV-C36 values were highest, with FIV-PCenv intermediate between FIV-C36 and FIV-PPR. However, the integrated copy number for the chimeric virus continued to rise and reached levels equivalent to FIV-C36 by day 180. Copy numbers for FIV-PPR also rose after an initial plateau between days 20–50, but remained approximately 1 log lower then FIV-C36 and FIV-PCenv throughout the experiment (Figure 5A). Mean proviral copies/10^6^ PBMCs were highest at 3 to 4 months PI. These values remained relatively constant for all groups as follows: FIV-PPR = 2.4×10^4^, FIV-PCenv = 1.14×10^5^, and FIV-C36 = 3.55×10^5^ (Figure 5A).

Similar trends were also reflected in bone marrow proviral loads, with FIV-PCenv levels remaining intermediate until time of the last biopsies, when they were equal to those of FIV-C36 (Figure 5B). Two FIV-PPR cats had undetectable bone marrow proviral FIV on day 17 PI; of these, one remained negative until between days 35 and 55 PI. One FIV-PCenv cat had undetectable bone marrow FIV at day 35 PI sampling (Table 2). Otherwise, all bone marrow samples tested were positive. Compared to PBMC levels, DNA copies/10^6^ bone marrow cells were higher on day 173 and 260 PI (Figure 5B and Table 2), and mean values were higher in FIV-PCenv than FIV-C36. Similar kinetics were noted in salivary viral RNA excretion, as follows.

**Table 2:** PBMC and bone marrow mean proviral loads.

	day 17 PI			day 35 PI		
	PBMC	BM	# cats BM+	PBMC	BM	# cats BM+
FIV-PPR	2.02E+03	3.21E+01	3/5	1.72E+02	8.01E+02	4/5
FIV-PCenv	2.52E+03	5.48E+01	5/5	2.27E+03	9.03E+02	4/5
FIV-C36	7.85E+04	9.27E+03	5/5	3.36E+04	9.13E+04	5/5

	day 55 PI			day 95 PI		
	PBMC	BM	# cats BM+	PBMC	BM	# cats BM+
FIV-PPR	1.81E+03	3.05E+03	5/5	2.40E+04	4.30E+02	5/5
FIV-PCenv	1.19E+04	2.56E+04	5/5	9.94E+04	4.25E+03	5/5
FIV-C36	1.03E+05	2.09E+05	5/5	2.43E+05	7.78E+04	5/5

	day 173 PI*			day 260 PI*		
	PBMC	BM	# cats BM+	PBMC	BM	# cats BM+
FIV-PPR	1.96E+03	4.52E+03	4/4	5.27E+03	7.95E+03	4/4
FIV-PCenv	1.61E+04	5.28E+04	4/4	4.44E+04	1.12E+05	4/4
FIV-C36	1.51E+04	4.00E+04	4/4	3.63E+04	7.29E+04	3/3

*One cat each from FIV-C36 and FIV-PPR groups were euthanized early due to severe allergies, while 4/5 samples were obtained from FIV-PCenv group on days 173 and 260 PI, and 3/4 from FIV-C36 group on day 260 PI, explaining lower number tested.

Saliva from FIV-PCenv infected animals was negative at time of first sampling (day 28), but rose steadily thereafter, reaching 1.9×10^4^ copies/ml by day 67 PI (Figure S1). FIV-C36 saliva viremia was higher than the chimera earlier in sampling, with detectable levels of 2.5×10^2^ copies/ml at first sampling, and reaching 4.76×10^4^ copies/ml by day 81 PI. Salivary viremia tapered after these initial peaks in both groups of animals, then increased again after day 120 PI, at which time FIV-PCenv levels exceeded FIV-C36. At time of last sampling (day 173 PI), FIV-PCenv mean saliva viremia was recorded as 6.31×10^4^ copies/ml, nearly four times that of FIV-C36 (1.75×10^4^ copies/ml) (Figure S1). Plasma and salivary viremia did not appear to be coordinated over time for either virus.

**Figure 6:**
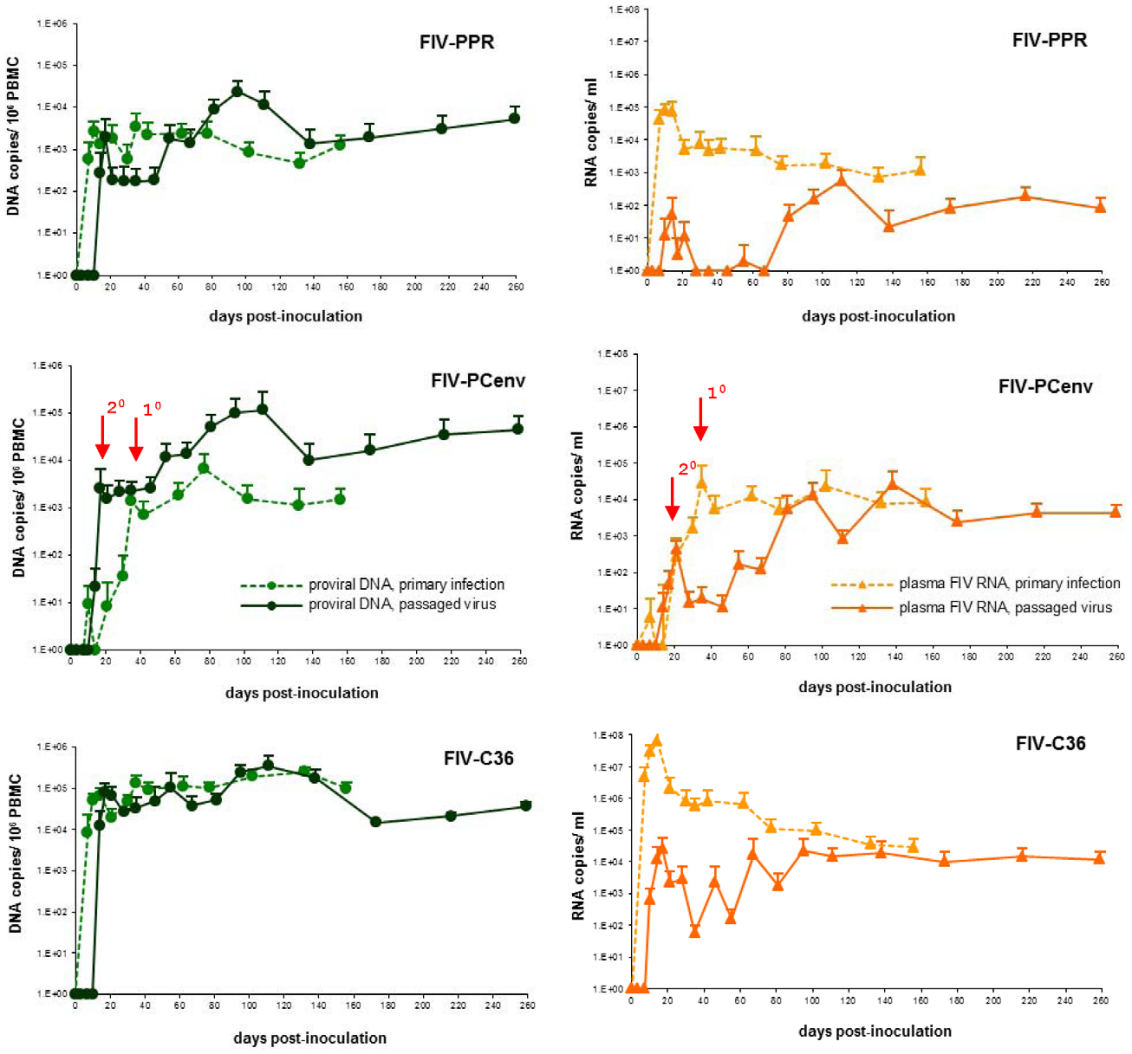
Comparative replication kinetics of primary (dashed lines) viral infection versus passaged (solid lines) FIV-PPR, FIV-PCenv, and FIV-C36. Proviral loads recorded as *gag* equivalents/10^6^ cells are depicted in green, and circulating virus as RNA genomes/ml in orange. Red arrows mark the delay in onset of acute viremia in primary versus secondary FIV-PCenv infections.

**Figure 7:**
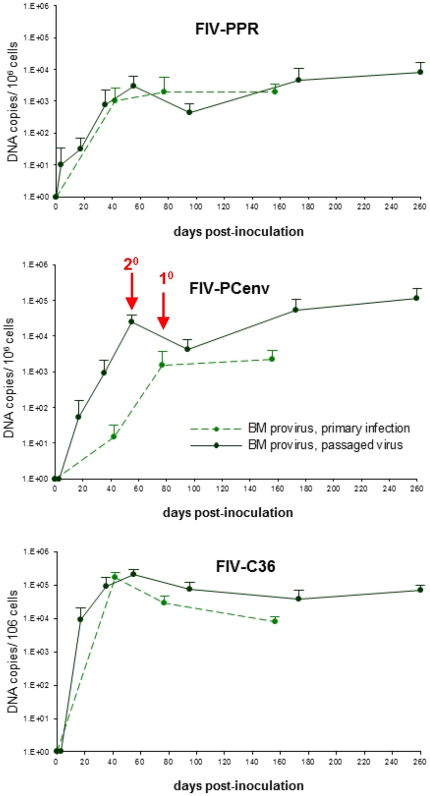
Comparative replication kinetics in bone marrow during primary (dashed lines) viral infection versus passaged (solid lines) FIV-PPR, FIV-PCenv, and FIV-C36. Proviral loads recorded as *gag* equivalents/10^6^ cells. Red arrows mark the delay in onset of acute viremia in primary versus secondary FIV-PCenv infections.

Comparisons were made of the time course for onset of acute viremia during primary infection versus results observed during secondary viral passage (Figure 6). Along with rescued replication kinetics from passage, FIV-PCenv PBMC proviral load was more than one order of magnitude higher than levels exhibited during primary infections described previously [24] in both PBMC (Figure 6) and bone marrow (Figure 7). This trend was not observed for FIV-C36 or FIV-PPR (Figure 6, 7). In contrast to trends for proviral load, plasma viremia levels tended to be lower for all three viruses during secondary infections (Figure 6). This is particularly true during days 1–60 for FIV-PPR and FIV-C36.

### Hematologic effects of passaged FIV-PCenv

**Figure 8:**
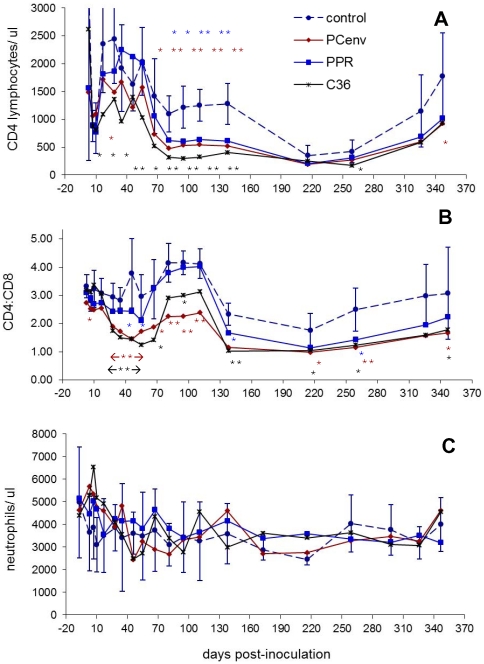
Timecourse of hematologic changes in domestic cats infected with passaged FIV-PPR, PC-env, and C36. Blood was sampled at various time points over the course of infection. Complete blood counts, differential leukocyte analysis, and CD4^+^ and CD8^+^ T-lymphocyte percentages in virus-inoculated cats (n = 5/group), or sham-inoculated control cats (n = 5) were calculated. CD4 counts (shown as number of cells/µl) (A), CD4∶CD8 ratios (B), and neutrophil counts (shown as cells/µl) (C) are demonstrated over the course of the study. For ease of viewing, error bars are shown for control group only. Statistically significant values relative to normal control values are indicated by asterisks in same colors used for each viral group (*, *P* between 0.01 and 0.05; **, *P*<0.01).

Onset of decreases in CD4^+^ T-lymphocyte relative to uninfected controls was similar to primary infections during FIV-C36 and FIV-PCenv infections; statistically significant differences between infected cats and non-infected controls were observed starting on days 17 and 63 PI, respectively, and persisted through day 138 PI (Figure 8A). FIV-PPR passage also resulted in significant decreases in CD4^+^ T-cell count starting at day 95 PI and persisting through day 138 PI. These declines were reflected in CD4∶CD8 ratios (Figure 8B).

Unlike initial infections, mean neutrophil counts for all infection groups remained above 2000 cells/µl (the baseline for feline neutropenia), with no significant differences when compared to non-infected control cats (Figure 8C). Individual animals from all infection groups and one negative control experienced intermittent neutropenia at some timepoints. FIV-C36 infection in particular resulted in neutropenia in three individuals starting at day 35 PI and persisting until day 111 PI whereupon the same three cats experienced intermittent neutropenia until day 259 PI (data not shown).

## Discussion

FIV infection of the domestic cat offers a model system for basic biological research of lentivirus-induced immunodeficiencies, along with development of treatments for HIV-AIDS. Two strains of FIV, FIV-PPR and FIV-C36, have been molecularly cloned and studied in relationship to severity of disease following productive infection in multiple laboratories [6], [23]. Higher viral titers and more rapid onset of clinical symptoms are consistently observed during experimental infections with FIV-C36 compared to FIV-PPR. FIV-C36, a highly pathogenic molecular clone of the clade C FIV isolate FIV-PGammar, differs in genetic sequence from the clade A molecular clone FIV-PPR by approximately 15% [6]. Therefore, it is rational to believe that infections of the domestic cat with molecular chimeras between FIV strains possessing differing pathogenic phenotypes can help identify which genetic elements contribute to progression to AIDS.

We have previously characterized viral kinetics and immunopathology resulting from primary infections with FIV-PCenv, a chimera containing the regulatory elements *vif*, *orfA*, *rev*1, and *env* from a highly virulent clade C strain (FIV-C36) on the background of a moderately virulent clade A strain (FIV-PPR). In preliminary studies [24], FIV-PCenv viremia kinetics displayed a lag period during the first month of infection, as well as a delay in classic indicators of immunodeficiency as reflected in drops in CD4^+^ T cell and neutrophil counts compared to parental viruses. Eventually, these parameters became intermediate to those of parental viruses.

Many studies have demonstrated that chimeras generated in the laboratory are typically less virulent than both parental clones [24], [29], [30], [31]. This is likely due to the fact that host innate and adaptive immune responses are mounted against viral infection, and successful isolates arise in the face of many factors designed to limit viral success. Thus, chimeric viruses can be crippled since portions of the virus have evolved independently, and adaptations in one portion of a wild-type virus may overcome detrimental mutations in another part of the genome. Therefore, it was striking that FIV-PCenv was able to recover a phenotype intermediate to parental viruses. Despite strong host immunity, a “diversity threshold” model has been proposed in which viral variants with beneficial mutations are able to persist and induce immunodeficiency when the number of diverse quasispecies is high enough [32]. This model has been supported by analysis of *env* evolution during rapid, serial passage of SHIVs in macaques [33]. During *in vivo* passage, the infecting virus pool has already overcome the diversity threshold in previous hosts. Thus, upon subsequent rounds of infection in naive hosts, there occurs faster onset of clinical disease accompanied by rapid antibody response and high viral loads [27]. The studies presented here sought to determine whether FIV-PCenv passage supported this selection hypothesis.

We surmised that chimeric FIV-PCenv may have accumulated mutations during the first-round of *in vivo* infection that allowed it to display a replication profile intermediate to parental viruses by day 77 PI. We hypothesized such changes would occur in the 3′ portion of the genome and were required to coordinate function between chimeric elements of the two parental viruses. However, analysis of 4200 nucleotides from the 3′ FIV-C36 half of the genome revealed no mutations in 6 FIV genome sequences compared to viral inoculum. Upon evaluation of the 5′ FIV-PPR half of the chimera, however, we identified an R813H Pol mutation in proviral sequences compared to viral inoculum in 6 PCR-derived clones from one infected individual, and confirmed by direct sequencing in another cat. Virus from the remaining three cats retained Arg (n = 1), or contained wild-type Cys (n = 2), as determined via direct sequencing of both integrated and circulating FIV-PCenv. This site in integrase typically has a conserved cysteine residue in domestic cat FIV, but varies in nondomestic cat FIVs and HIV. This finding suggests that during *in vitro* passage, a T→C mutation arose in the TGT codon for cysteine in parental PPR leading to CGT (arginine) in the inoculum. Following passage in two animals, a G→A mutation was introduced, resulting in a replacement of arginine with histidine (CAT). Virus containing cysteine that was recovered from two cats may represent a minor population present in inoculum undetected during sequencing of clones, or a reversion to wild-type due to pressure at a mutation hot-spot. The fact that this site is not occupied by cysteine in 3 nondomestic FIVs, or by homology, HIV indicates that it is not conserved across all lentiviral genomes.

Change in a glutamic acid residue highly conserved among lentivirus species, and positioned four amino acids upstream of Pol C813 in FIV-PCenv (Figure 2, red highlight), confers resistance to the integrase inhibitors raltegravir and elvitegravir [34]. Further, FIV Pol C813 may be a component of secondary structural motifs important for integrase function, as this region is part of alpha-helix-1 in HIV [35], along with SIV [36], primate foamy virus [37], and Rous sarcoma virus [38] integrases. In addition to being proximal to a potential target for drug-resistance mutations, a panel of synthetic peptides containing the HIV homologue to FIV Pol C813 (HIV Pol E811) were shown to stimulate IFN-γ production from CD8^+^ T cells [39]. Together, these reports suggest that this area of integrase is conformation flexible, and serves as a target for both drug resistance and the immune response; our findings here would be consistent with escape from immunological pressure. Additional studies will determine if CTL haplotypes in PBMC from FIV-PCenv infected cats matches peptides containing Pol 813.

It is interesting that we observed nonsynonomous mutations at site Pol 813 in four of five animals following the apparent initial nonsynonomous mutation which occurred in viral inoculum. This would suggest this residue was under significant mutational pressure both *in vitro* and *in vivo*, and likely accounts for the delayed replication kinetics noted during initial viral infection. Indeed, animals harboring either an Arg or His mutation had higher viral loads than cats in which wild-type Cys was detected (Figure 3). Because FIV-PCenv contains approximately 200 residues of FIV-PPR integrase, with the remaining 80 residues from FIV-C36, it is possible that important interactions occur between residue 813 (occurring in the FIV-PPR portion of the genome) and residues/structural components of the terminal ∼30% of the protein. Evaluation of genomes rescued from cats infected with passaged virus, in which the R813H substitution was uniformly found, supports this hypothesis, and suggests that (1) delayed viral replication kinetics during primary infection resulted from R813H, restoring replicative capacity following *in vitro* mutation, and (2) FIV-PCenv virulence results from 3′ elements of FIV-C36 which were not measurably altered during *in vivo* passage. Since pooled plasma from the first group of animals was used as material for viral passage, and Cat# FIV-PCenv-1 had the highest viremia, it is not surprising that we detected this strain in all animals from the second group. What is noteworthy; however, is the fact that R813H may have played a mechanistic role in the rescue of replication kinetics. It would be interesting to evaluate whether genomic substitution restoring the original FIV-PPR sequence at this site (H813C) would additionally enhance FIV-PCenv virulence, and may explain the observation of cysteine at this site in two cats from the first study.

While plasma viremia in FIV-PCenv cats mirrored that of FIV-C36 following primary infection, proviral copy numbers in PBMC or bone marrow of cats infected with the chimera never reached the values of FIV-C36 [24]. However, *in vivo* passage of FIV-PCenv resulted in viral RNA and DNA copy numbers equal to those measured for passaged FIV-C36 by day 81 post-inoculation both peripherally and in bone marrow and saliva (Figures 6, 7, S1). Moreover, FIV-PCenv proviral loads were one order of magnitude higher upon passage, suggesting that either the initial replication-competent FIV-PCenv inoculum dose was higher for second round infections, or that progressive replication enhancement continued during viral passage.

The reason for lower plasma viral loads following exposure to primarily passaged FIV-PPR- and FIV-C36-containing plasma is unknown. Primary inoculations were composed of tissue culture supernatant whereas the secondary challenge utilized pooled plasma from cats. It is possible that plasma factors inhibited initial viral replication, or that quasispecies transmitted had lower viral replication rates. Plasma inoculum was normalized to equalize viral input based upon real-time PCR measurements of particles/ml. Because peak viral load during initial inoculation occurred earlier during FIV-PPR and FIV-C36 infection (day 14 versus day 35 for FIV-PCenv), inoculums were prepared during different stages of infection. Because FIV-PCenv peak plasma viremia was lowest during primary inoculation, FIV-PPR and FIV-C36 inocula were diluted with naïve pre-inoculation plasma from the same cats, whereas FIV-PCenv plasma was used undiluted. It is possible these variations in inocula resulted in inaccuracies in exposure, or presence of quasispecies with differing virulence characteristics.

Although neutropenia was observed in three FIV-C36 cats between days 35 and 259 PI, mean neutrophil levels never significantly varied from those of mock-infected controls. This may have related to the fact that control cats had low neutrophil counts at seven time points; factors such as cage environment, allergies, or stress can contribute to variability in hematology parameters [40]. One FIV-PCenv cat also experienced neutropenia from day 46–95 PI, and two different FIV-PPR cats experienced neutropenia on days 3 and 173 PI, respectively. None of these effects, however, resembled the marked neutropenia observed during first-round infections with FIV-C36 or FIV-PCenv. The more dramatic neutrophil declines observed during primary infections with FIV-C36 and FIV-PCenv may be related to factors associated with use of a biological inoculum (pooled plasma), or the fact that the inoculum contained more varied viral quasispecies, and parallels the observed lower plasma viremia established in this experiment.

Similar to primary infections, CD4^+^ T-cell declines were observed starting on day 17 PI in FIV-C36 cats which persisted until day 138 PI. Statistically significant differences in CD4^+^ T-cell counts of the FIV-PCenv group compared to controls began on day 63 PI. Previous infections with this chimera resulted in a rebound in CD4 levels compared to controls. Conversely, during second-round infection, *P* values remained below 0.01 through day 138 PI. The FIV-PPR group also had significant differences compared to controls between days 95 and 138 PI, strengthening the hypothesis that viral passage results in enhanced virulence.

In this study we were able to evaluate distribution of proviral DNA in bone marrow and compare viral RNA load obtained from saliva with plasma viremia. These measures re-iterated kinetics noted peripherally; namely, FIV-C36 initially peaked at loads above FIV-PPR and the chimeric construct, but after approximately 81 days PI, FIV-PCenv levels exceeded those of parental viruses. Because receptor-viral interactions are mediated by 3′ *env* determinants represented by FIV-C36 in the chimeric virus, we reasoned that the distributions of the viruses might vary if Env tropism dictated differential cell susceptibilities. The data generated in this study would suggest that replication competency and virus shedding in saliva is more related to overall replicative capacity versus Env-restricted tissue targeting, though a role for enhanced replication in circulating cells of the bone marrow or lymphoreticular system conferred by the 3′ end of FIV-C36 cannot be discounted by this study.

The findings presented here indicate a role for FIV-C36 elements in FIV-PCenv pathogenesis compared to FIV-PPR infections. To determine whether a particular genomic element is responsible for the heightened virulence of FIV-C36 versus bulk 3′ viral genomic substitution, it will be necessary to perform infections using chimeras with single 3′ FIV-C36 genes on the FIV-PPR background. Experiments with replication-competent FIV accessory-gene chimeras in which smaller regions of the genome have been substituted would provide more insight into specific genetic factors that influence viral replication rates and virulence. Perhaps the most interesting finding reported here is the association of enhanced replicative capacity *in vivo* with rescue of a mutation which apparently arose during *in vitro* replication. This study would suggest that residue 813 in FIV Pol is essential in conferring *in vivo* replication, but is apparently not essential for *in vitro* replication. Enhanced pathogenicity of chimera FIV-PCenv relative to parental strain FIV-PPR that was independent of mutations in 3′ FIV-C36 elements suggests that *env* and 3′ regulatory elements in FIV determine strain-dependent pathogenicity.

## Materials and Methods

### Ethics Statement

This study was approved by the Colorado State University Institutional Animal Care and Use Committee, “Molecular Analysis of a Highly Pathogenic FIV”, 07-125A. Colorado State University's animal care program is licensed by the USDA, accredited by AAALAC, Intl, and holds an OLAW assurance (A3572-01).

### Cloning and analysis of FIV-PCenv sequences

FIV-PCenv stock was used as previously described [24] to infect Cat#s FIV-PCenv-1 through 5 both orally and intravenously with 10^3.5^ TCID_50_ particles/ml total (0.5 ml each route). Genomic DNA was extracted from PBMCs purified on a Histopaque-1077 (Sigma) gradient. For whole genome sequencing, day 77 PI from Cat# FIV-PCenv-1 was chosen as it represented the highest FIV-PCenv proviral load attained during the study (1.68×10^4^) [24]. It was reasoned that the greatest likelihood of detecting nucleotide differences compared to inoculum would be in this sample. Cells were washed and pellets frozen at −20°C overnight. DNA was isolated using a DNeasy Tissue Kit (Qiagen). PCR conditions were 30 cycles of denaturation at 94°C for 1 min, annealing at 57°C for 30 s, and extension at 71°C for 1 min. Primer sequences were as follows:

fA.1 = CCGAACAGGGACTTGAGAAAG, rA.1 = TGGTGCAAATCTTGCTTCTG,
fA.2 = CACCTACTGACATGGCCACA, rA.2 = GGTGAGGTAGTCCCAACTG,
fA.3 = AAGTGGAAAATGGAGGATGC, rA.3 = CTTCTTGCCAGATTCCTTCC,
fA.4 = AGAAGGCGGAAATACAAGCA, rA.4 = TCTTCACTCATCCCCTTCAG,
fC.1 = GGGTAGAATAGGGGGAATGG,
rC.1 = GCCTTACCTTGTCCTGCATA,
fC.2 = CCAGAAGAGGCAGAGGAATT,
rC.2 = CTGTTCCTGCTCCTGCAATG,
fC.3 = CAACAGATTGGGGTTACATG, and
rC.3 = TGAGTCATGTTCAGCTGTTTCC.

Products were separated on an agarose gel containing ethidium bromide, excised, and purified using the QIAquick gel extraction and purification kit (Qiagen). We used TA cloning with pPCR2.1 vector (Invitrogen) to propagate FIV-PCenv sequences in Top10 *E. coli*. Colonies were screened via restriction enzyme and PCR analyses, and Qiagen mini-prep kits used to purify plasmid DNA. Viral inoculum was similarly amplified and evaluated; cDNA was generated using RNA purified from FIV-PCenv viral stock (QIAamp Viral RNA Mini Kit, Qiagen) as template in reactions with SuperscriptII (Invitrogen) reverse transcriptase. Consensus sequence was obtained from six-eight clones per region amplified. Direct sequencing of genomic DNA and viral cDNA for all Phases I and II individuals was performed with reverse primer TCCAAGGAGGTTGTTTCAGG on gel purified templates amplified via nested PCR with outer primers fA.4/rA.4 and inner primers fA.4-2 = CAAGATGGTGGCAGAAGAGA/rA.4-2 = GGCCATCCCTCCTATTCTA. Sequencing was performed at TSRI Center for Nucleic Acid Research (TOPO clones C36 region) and The University of Nebraska's Center for Virology (TOPO clones PPR region and all direct sequencing). Nucleotide and amino acid alignments were analyzed with Sequencher™ (Gene Codes Corporation, Ann Arbor, MI) and VectorNTI (Invitrogen).

### *In vivo* infections

Twenty 14–16 week-old, SPF cats were housed in gang rooms in AAALAC-international accredited CSU animal facilities following protocols approved by CSU ACUC. Three groups (n = 5) of unanesthetized cats were inoculated intravenously with 1 ml pooled plasma from five cats previously infected with either FIV-PPR, FIV-PCenv, or FIV-C36. Plasma from time point of peak viremia for each virus (day 14 post-infection for FIV-PPR and FIV-C36; day 35 for FIV-PCenv, based on RT-PCR values) was normalized using pre-inoculation naïve plasma from matched animals so that all animals received an equal number of viral particles (1.38×10^4^). Five cats were administered naïve plasma and used as non-infected controls. Animals were monitored daily for clinical signs of illness throughout the study, and physical exams were performed at blood collection. Weight measurements and blood collections were performed on days −7, 3, 7, 10, 14, 17, 21, 28, 35, 46, 55, 67, 81, 95, 111, 138, 173, 216, and 259 PI for detection of circulating virus and PBMC provirus, along with hematologic analysis described below. One cat from each FIV-PPR and FIV-C36 groups were euthanized early prior to day 173 PI due to obvious allergic dermatitis apparently unrelated to FIV infection. Bone marrow samples were collected from the humerus following ketamine/acepromazine/burtorphanol anesthesia on days 3, 17, 35, 55, 95, 173, and 260 PI and were analyzed for provirus.

### Hematologic analysis

To determine percentage of cells positive for CD5, CD4, and CD8 surface antigens, 30 µl of EDTA anti-coagulated blood was added to 12×75 mm polystyrene tubes, along with 0.15 µl of Alexa647-labeled anti-CD5, 0.15 µl of FITC-labeled anti-CD4, and 0.3 µl of PE-labeled anti-CD8 mouse monoclonal antibodies (Southern Biotech) diluted in FACS buffer (5% BSA, 0.1% sodium azide in PBS). Anti-CD4 was directly labeled with FITC, anti-CD8 to PE, and anti-CD5 was unlabeled but subsequently conjugated to Alexa647 using a Zenon kit (Invitrogen). Samples were incubated in the dark at room temperature with rocking for 30 min, washed 2×, diluted to 100 µL with 1× PBS +2% BSA. Next, red blood cells were lysed, and stained cells were fixed using a Beckman Coulter Q-Prep work station with 600 µl of 0.1% Formic Acid, 270 µl of 0.06 M Na_2_CO_3_ anhydrous, 0.25 M NaCl, 0.25 M Na_2_SO_3_, and 90 µl 1% wt/vol paraformaldehyde in 1×PBS. Flow cytometry was performed on a CyAn cell sorter (Dako Cytomation), and results analyzed using Summit software package (Dako Cytomation). To determine complete leukocyte and RBC counts from EDTA blood, a Z1 Series Coulter Counter was used. Differential leukocyte counts were determined manually from stained smears. Absolute neutrophil and lymphocyte counts were calculated by multiplying the total leukocyte count by the percentages of neutrophils or lymphocytes for each cat at each time point. To determine absolute CD5^+^, CD4^+^ and CD8^+^ cell counts, total lymphocyte counts were multiplied by percentage of Alexa647 (CD5), FITC (CD4), or PE (CD8) fluorescing cells. Blood was collected from all cats prior to infection to establish baseline values.

### FIV proviral DNA and plasma RNA quantitation

Plasma was collected from EDTA-treated whole blood following centrifugation and frozen at −70°C until processing. RNA was purified from 140 µl of plasma using a QIAamp Viral RNA Mini Kit (Qiagen). SuperscriptII (Invitrogen) was implemented in reactions with random hexamers (Invitrogen) added, and treated with RNase Out (Invitrogen) for preparation of cDNA from viral RNA. Genomic DNA was extracted from PBMCs purified on a Histopaque-1077 (Sigma, St. Louis, MO) gradient. Cells were washed and pellets frozen at −20°C overnight. DNA was isolated using a DNeasy Tissue Kit (Qiagen).

Real-time PCR reactions were run using an iCycler thermocycler (Bio-Rad) to detect both proviral and circulating FIV *gag* using the AmpliTaq Gold DNA polymerase-containing TaqMan Universal PCR Master Mix (Applied Biosystems). Sequences of primer/probe (5′FAM, 3′TAMRA) sets are reported in [22] and were as follows: FIV-A, f = GCCTTCTCTGCAAATTTAACACCT, r = GATCATATTCTGTCAGTTGCTTT, and p = TGCGGCCATTATTAATGTGGCCATG. FIV-C, f = ACTCACCCTCCTGATGGTC CTA, r = TGAGTCAGCCCTATCCCCATTA, and p = ACCATTGCCATACTTCACTGC AGCCG. PCR reactions in a total volume of 25 µl consisted of 12.5 µl master mix, 0.5 µl each of 20 µM forward and reverse primers, 0.2 µl of 10 µM probe, and 5 µl template. After 2 min at 50°C, the AmpliTaq Gold DNA polymerase was activated at 95°C for 10 min, followed by 45 cycles of 95°C for 15 s and 60°C for 1 min.

Threshold cycle values (CT) were defined as the point at which the fluorescence passed a threshold limit. Copy number for FIV provirus was calculated using a standard curve generated from dilutions of a sub-cloned *gag* PCR product. To calculate copy number of viral RNA in plasma, a standard curve was generated by diluting FIV-PPR virus stock in naïve cat plasma; this was prepared and analyzed by reverse-transcriptase quantitative PCR as described above. CT values were compared to those of the sub-cloned *gag* standard to assign values. Lower limits of detection approached 10 RNA or DNA equivalents. Characteristics of samples in this range included CT values over 40, higher standard deviation between replicates, or detectable signal in only one or two of three replicates.

Salivary viral RNA quantitation was similarly performed and is described in detail in Supporting Information S1.

### Statistics

To analyze decreases in CD5, CD4, and neutrophil counts, and CD4∶CD8 ratios for infection groups compared to controls at each time point, GraphPad Prism® was used to determine one-tailed *P* values in an unpaired t test. *P* values below 0.05 were considered significant.

## Supporting Information

Figure S1 **Salivary viral loads from clade C FIV-C36, and A/C chimeric FIV-PCenv infected cats.** Group averages with standard deviation relative to plasma viremia (A). Trends for saliva and plasma RNA levels over time (B) presented as RNA copies/ml. Mock-infected cats had undetectable viremia (data not shown).
(TIF)

Supporting Information S1 Salivary viral RNA quantitation.
(DOC)
