# Supplementary figures and images for: Pathogenicity and Rapid Growth Kinetics of Feline Immunodeficiency Virus Are Linked to 3′ Elements
Source: PLoS One. 2011 Aug 26;6(8):e24020. doi: 10.1371/journal.pone.0024020 (PMC3162592; doi:10.1371/journal.pone.0024020)

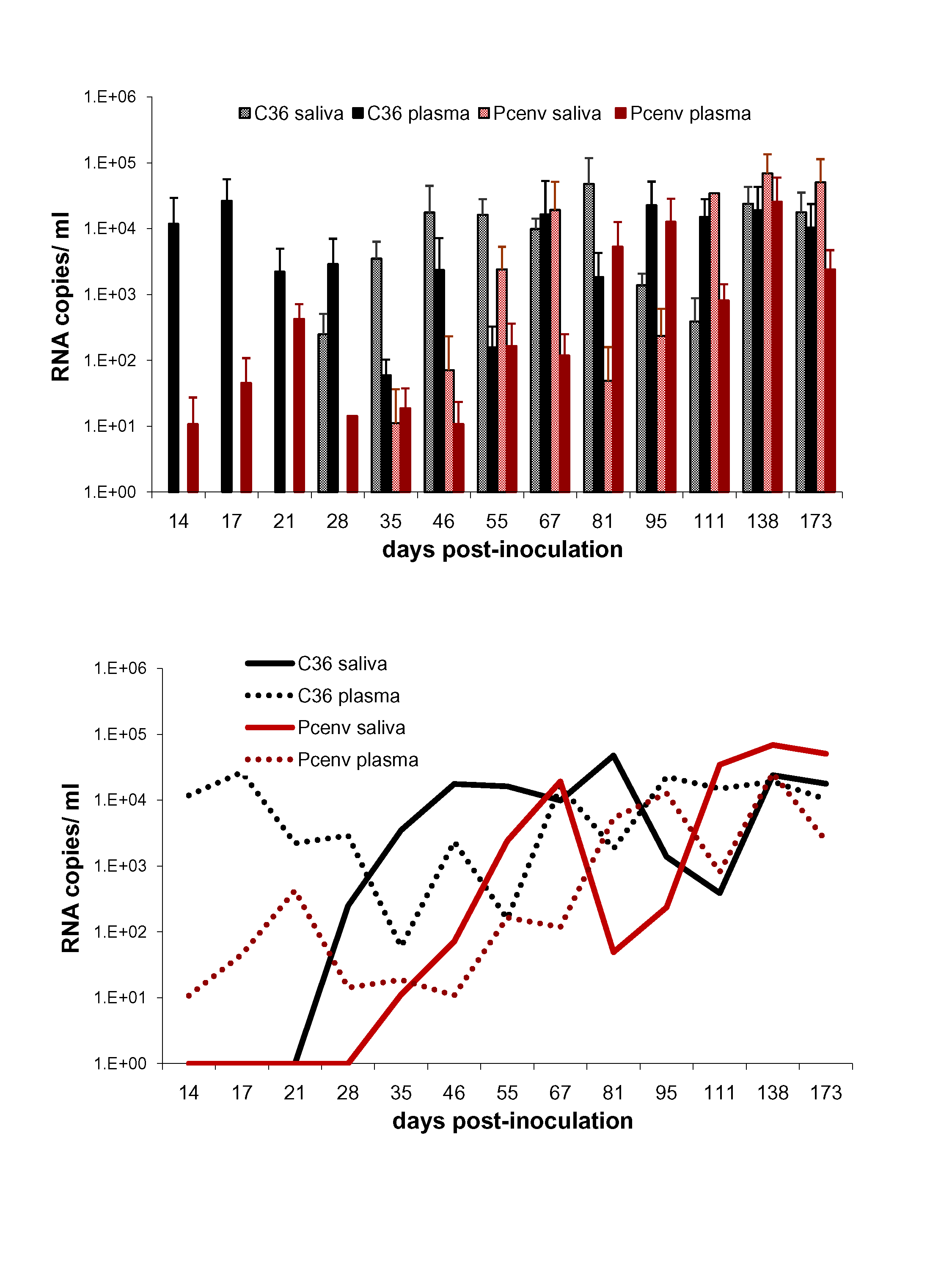

Supplement: Figure S1 — Salivary viral loads from clade C FIV-C36, and A/C chimeric FIV-PCenv infected cats. Group averages with standard deviation relative to plasma viremia (A). Trends for saliva and plasma RNA levels over time (B) presented as RNA copies/ml. Mock-infected cats had undetectable viremia (data not shown). (TIF) [file pone.0024020.s001.tif]
