# Supplementary material for: Pathogenicity and Rapid Growth Kinetics of Feline Immunodeficiency Virus Are Linked to 3′ Elements
Source: PLoS One. 2011 Aug 26;6(8):e24020. doi: 10.1371/journal.pone.0024020 (PMC3162592; doi:10.1371/journal.pone.0024020)
Supplement: Supporting Information S1 — Salivary viral RNA quantitation. (DOC) [file pone.0024020.s002.doc]

**Supporting Information**

Salivary viral RNA quantitation

Saliva was collected on days 28, 35, 46, 55, 67, 81, 95, 111, 138, and 173 by swabbing buchal mucosa. Swabs were placed into 1.5 ml tubes and centrifuged to collect liquid for RNA extraction and cDNA synthesis. Saliva was obtained from under the tongue and cheek pouches of each cat using a sterile cotton swab. Wetted swabs were immediately broken off into 200 µl of RNA*later* Solution (Ambion, Austin, TX) and stored at -20^o^C until processing. Stored swabs were thawed at room temperature, vortexed vigorously for 1 min, and centrifuged at 2000 rpm for 1 min. To collect saliva from the swab tip, swabs was inverted using sterile forceps washed with 70% ethanol between samples, spun at 2000 rpm for 2 min, then discarded. Viral RNA was extracted using the RNAqueous total RNA isolation kit (Ambion, Austin, TX), according to manufacturer’s instructions. Samples were eluted in 50 µl, and ethanol precipitated (2.5*vol 100% EtOH, 0.1*vol 3M sodium acetate, and 1 µl glycogen) overnight at -20^o^C. Precipitated RNA was pelleted at 14K rpm for 20 min at 4^o^C and resuspended in 20µl of RNA Storage Solution (Ambion, Austin, TX).

RNA from each sample was used as template to synthesize cDNA using the RETROscript reverse transcription kit (Ambion, Austin, TX). The total volume of extracted RNA was transferred into two 20 µl reactions with random decamer primers and following manufacturer’s instructions for RT without heat denaturation of RNA. Each sample was analyzed by real-time PCR in triplicate using an iCycler thermocycler (Bio-Rad, Hercules, CA) with reaction components and cycling parameters previously described [24]. Primers and probes used in PCR reactions have been previously characterized [22]. To quantitate viral copy number in each reaction, a six-point standard curve was generated by diluting virus stock in a 10-fold dilution series into RNA*later* solution. Each dilution was then extracted and used as template in cDNA reactions as described above, and assigned a copy number value based on comparison to a *gag* plasmid standard curve ranging from 10^5^ to 10^1^ copies per reaction. A threshold was set according to the run data for each plate, and CT values greater than those of negative controls were included in analysis.

The resulting copy number data for each sample was analyzed using GraphPad Prism® software (La Jolla, CA). Triplicate values for each sample were averaged, and calculated to determine viral copies per ml saliva. Standard errors were calculated for each treatment group at each timepoint.
